# The Healthiest Capital in the World

**Published:** 1894-10

**Authors:** 


### The Healthiest Capital in the World.

Judged by its death-rate the health of London compares most favorably with that of any other large city, at home or abroad. The recent weekly statistics of the Registrar-General show that the metropolis is now remarkably healthy, and the death-rate almost the lowest on record. During the last four weeks, for which returns are available, the London mortality has been equal to an annual rate of only 16.3 per 1,000 persons living, while in Paris it was 20.5, in Berlin 18.2, in Vienna 22.5 and in New York 19.6. The death-rate in London is now considerably lower than in any foreign city containing a population of half a million or upward ; and with one or two exceptions actually lower than in any other of the thirty-one large colonial and foreign cities for which returns are published by the Registrar-General. On only three previous occasions on record has the London death-rate been so low as during the current month, and in every week since January last the deaths have been considerably below the average. Although during the quarter just ended the mortality from the principal zymotic diseases in London has exceeded the average, owing to the prevalence of measles and diphtheria, the meteorological conditions have generally been unusually favorable to the public health. The mortality from diseases of the respiratory organs has been considerably below the average each week throughout the quarter, and the cool weather has delayed the appearance of summer diarrhea, which invariably causes a rapid rise in the death-rate about this period of the year.—*British Med. Journal.*

